# Spontaneous complete uterine rupture with protrusion of foetal limbs at the third trimester following laparoscopic cornuostomy: A case report

**DOI:** 10.1097/MD.0000000000028955

**Published:** 2022-02-25

**Authors:** Jianyang Feng, Yahui Kang, Guixian Chen, Yaoyue Zhang, Yuan Li, Yi Li, Hong He

**Affiliations:** a Department of Obstectrics and Gynecology, The Third Affiliated Hospital of Guangzhou Medical University, Guangzhou, Guangdong, China; b Department of Gynecology, Gansu Provincial Maternity and Child-Care Hospital, Lanzhou City, Gansu, China; c Department of Gynecology and Obstetrics, The People's Hospital of Nanchuan, Chongqing City, China; d Department of Gynecology, Fengcheng Maternity and Child-Care Hospital, Yichun City, Jiangxi, China.

**Keywords:** laparoscopic cornuostomy, spontaneous uterine rupture, third trimester

## Abstract

**Rationale:**

Spontaneous complete uterine rupture during gestation is rare and has no specific symptoms; however, it is a life-threatening event for both the fetus and mother. The rupture typically happens in labor and is uncommon before labor. Herein, we present the case of a woman, encountering complete rupture at third trimester followed by laparoscopic cornuostomy.

**Patient concerns:**

A 26-year-old woman presented with acute right lower abdominal pain at 33 weeks and 5 days of gestation.

**Diagnoses:**

We made a diagnosis of threatened uterine rupture.

**Intervention:**

Urgent cesarean section performed. Exploration of the uterine dehiscence wound demonstrated that the myometrium was completely ruptured at the primary laparoscopic surgical scar with a defect of 40 mm, and live birth and preservation of the uterus was achieved.

**Outcome:**

On the third day of operation, she had a good recovery and was discharged. After a 6-week postpartum follow-up, she displayed a good level of rehabilitation.

**Lessons:**

Pregnancy after laparoscopic cornuostomy should be treated as high-risk gestation and the rupture during gestation of the uterine scar should be suspected once lower abdominal pain occurred. Swift diagnosis and prompt intervention play a crucial role in saving the lives of the fetus and the mother.

## 1 Introduction

Uterine rupture refers to full-thickness destruction of the uterine myometrium and overlying serosa. Uterine rupture during gestation is rare and has nonspecific symptoms; however, it is a life-threatening event for both the fetus and mother. One of the main risk factors that has been identified is iatrogenic myometrial incision by laparoscopy or laparotomy penetrating the uterine cavity. Laparoscopic cornuostomy, conducted to remove the pregnant lesion via cornual hysterotomy, is one of the procedures for management of cornual ectopic pregnancy.^[1,2]^ Compared with traditional laparotomic approach, laparoscopic cornuostomy requires more learning-curve time and practical suturing experience. And the justice of laparoscopic approach in the management of cornual ectopic pregnancy had not been verified. Limited evidence from previous studies indicated that the risk of uterine rupture after cornual pregnancy following laparoscopic surgery was higher than laparotomic.^[3,4]^ However, these was no specific and sensitive manifestations to predict the rupture during pregnancy followed by laparoscopic cornuostomy. Thus, pregnancy after laparoscopic cornuostomy should be treated as high-risk gestation and the rupture during gestation of the uterine scar should be suspected once lower abdominal pain occurred. Reliable and layered suturing of the myometrium is crucial to repair the ruptured defect intraoperatively.

The fetal–maternal outcome following uterine rupture depends on prompt diagnosis and rapid intervention. However, the symptoms are not specific and there are no standard measurements to precisely predict uterine rupture following cornual incision.

Herein, we present the case of a patient who experienced spontaneous symptomatic complete rupture of the uterus with cornual scarring with intact amniotic sac and foetal limb protrusion out of the peritoneal cavity at 33 weeks and 5 days of gestation.

## 2 Case report

**Figure 1:**
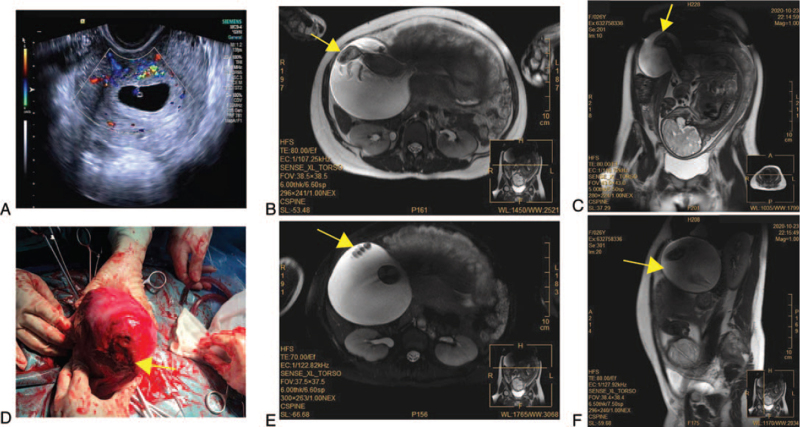
Ultrasound, magnetic resonance imaging, and surgical image of the patient. (A) Ultrasound demonstrated a gestational sac implanted in uterine cavity in the first trimester. (B-C and E-F) MRI demonstrated a dehiscence at the right aspect of the uterine horn with hernial sac containing amniotic fluid and foetal lower limbs protruding out of the peritoneal cavity. (D) The myometrium was completely ruptured at the primary surgical scar.

A 26-year-old woman, G2P0, presented to the emergency clinic in November 2020 complaining of right lower abdominal pain for the past 8 hours. She was at 33 weeks and 5 days of pregnancy. Her last menstrual period was on February 29, 2020. An ultrasound test in the first trimester showed the gestational sac to be embedded in the uterine cavity (Fig. 1A). There was no prior trigger or history of trauma. The pain was moderate, persistent, fixed, and tolerable. No fever, vaginal bleeding, or fluid release or other gastrointestinal symptoms were observed. Fetal movement was normal. She gave a history of laparoscopic cornuotomy performed at our center at the 9th week of a cornual pregnancy as a primigravida, after which she was recommended contraception for 12 months. She had no history of liver, kidney or autoimmune disease, nor any history of urolithiasis. There was nothing suggestive or significant about her family history.

At admission, a physical examination was routinely performed. Blood pressure was 113/65 mmHg, heart rate 80 beats per minute (bpm), respiratory rate 20 bpm, temperature 36.7°C and fetal heart rate (FHR) 142 bpm. Cardiopulmonary auscultation revealed nothing suggestive. Abdominal palpation indicated a right lower abdominal fixed pain around the right uterine fundus without rebound tenderness. Murphy's sign was negative and percussion of renal regions on both sides revealed no pain or other signs. No positive physical sign of ureteralgia was detected. The uterine contraction was unpalpated. The estimated fetal position was left occipitoanterior. The nonstress test on the fetus was reactive. Progestation body mass index was 17.6 kg/m^2^.

Routine blood tests indicated hemoglobin at presentation was 124 g/L, white blood cell counts 13.98 × 10^9^ cells/L, platelet counts 183 × 10^9^/L (reference range 115 to 150 g/L, 3.50 to 9.50 × 10^9^/L, and 125 to 350 × 10^9^/L, respectively). Investigations for coagulation, demonstrated thrombin time (TT) 13.6 s (10 to 17 s), prothrombin time (PT) 10.1 s (8.8 to 13.8 s), activation partial thrombin time (APTT) 26.9 seconds (28–42 seconds), and fibrinogen 3.88 g/L Tests for kidney and liver function demonstrated blood creatinine (Crea) 40 μmol/L (41–73 umol/L), serum alanine aminotransferase (ALT) 14.9 U/L (7–40 U/L), serum albumin (ALB) 34.0 g/L (35–50 g/L), total bilirubin 4.2 μmol/L (0–21.0 μmol/L), respectively.

Possible differential diagnoses for the patient included ovarian cyst/tumor torsion, placenta percreta implantation, concealed placental abruption, threatened or complete uterine rupture, acute appendicitis, urolithiasis, and other nonobstetrical pathologies. Based on the symptoms, history of laparoscopic cornual hysterotomy, and the findings of the physical examination, a diagnosis of uterine rupture was suspected, but gestational acute appendicitis and concealed placental abruption could not be excluded completely. Because of the difficulties and disadvantages of ultrasound in detecting gestational acute appendicitis, a magnetic resonance imaging (MRI) study was recommended. Therefore, urgent unenhanced abdominal MRI was performed and it revealed a 49 mm dehiscence (Fig. 1B) at the right of the uterine horn with a 117 × 116 mm hernial sac containing amniotic fluid and foetal lower limbs (Fig. 1C, E). We suspected a complete rupture of the uterine myometrium with intact amniotic sac protruding out of the peritoneal cavity (Fig. 1F). During the diagnostic workup, vital signs and FHR were stable.

Consequently, the rapid response team was mobilized, including the department of anesthesiology, blood transfusion, neonatal pediatrics, and obstetrics, and urgent lower uterine segment cesarean section was organized. The patient and her husband were informed about the possible complications of the surgery in detail, including the possibility of hysterectomy and loss of the fetus. The operation was started with a longitudinal lower abdominal incision. During the cesarean section, only a small amount of bloody fluid was observed in the abdominal cavity. Within 2 minutes after the incision, a premature live male was delivered, weighing 2000 g with Apgar scores of 8, 9, and 10 at 1, 5, and 10 minutes, respectively. Exploration of the uterine dehiscence wound showed that the myometrium was completely ruptured at the primary surgical scar with a defect of 40 mm (Fig. 1D). Then 2-layer uterine closure was done using full-thickness continuous suture by 1-0 (CL-923) and 2-0 (CL-923) polysorb (COVIDIEN), respectively. The uterine myometrium and serosa were separately repaired. The interstitial section of the right fallopian tube was also involved and damaged. Thus, right salpingectomy was also performed. Oxytocin, hemabate (carboprost trometamol), and ergonovine maleate via myometrial injection were prescribed intraoperatively to promote uterine contraction and decrease blood loss, respectively. The estimated total blood loss was 400 mL. Oxytocin was maintained for 6 hours intravenously.

Postoperatively, Latamoxef was administered every 12 hours for a total of 48 hours for prophylactic purposes. The first day after the operation, hemoglobin was 107 g/L. Ultrasound scan for uterus and adnexa displayed no abnormality. Two days later, on the third day of operation, she had a good recovery and was discharged. After a 6-week postpartum follow-up, she displayed a good level of rehabilitation. This report had been approved by the Hospital Ethics Committees.

## 3 Discussion

Spontaneous antenatal uterine rupture is an uncommon but potentially fetal–maternal life-threatening event during gestation.^[5,6]^ A history of uterine surgery has been reported as the most common risk factor for spontaneous rupture in retrospective studies.^[3,6]^ Laparoscopic hysterotomy was associated with a higher risk of uterine rupture than laparotomic hysterectomy.^[3]^ In their study, Gil et al^[3]^ reported that uterine ruptures occurred in 0.43% (237/54,146) of patients following myomectomy, and the number of uterine ruptures per 1000 myomectomies were 4.2 after laparotomy versus 10.6 after laparoscopic procedures. Chao et al^[4]^ retrospectively retrieved information on 20 cases of uterine rupture from their center over 15 years, and found that 13 noncesarean scar ruptures were identified in women with a previous history of laparoscopic approaches to the uterus, including 76% that occurred after laparoscopic myomectomy, 8% following hysteroscopic myomectomy, and 16% after laparoscopic wedge resection of cornual ectopic pregnancy.

Cornual pregnancy is a rare occurrence in ectopic pregnancy. However, there is no consensus on the duration of contraception needed after surgery for cornual pregnancy. Recently, Arakaki et al^[7]^ reported a case of a patient who experienced threatened uterine rupture at 16 weeks of gestation; this was suspected by MRI scanning with a 1.5-month contraception after laparoscopic cornuostomy. As in our case, typically spontaneous uterine scar rupture occurred at 33 weeks of gestation with more than a year's duration of contraception after laparoscopic cornuostomy. A study conducted by Jansa et al^[8]^ reported 4 patients who experienced uterine rupture after hysteroscopic septum resection; the median time to pregnancy among them was 17 months (range 1–60) with acceptable foetal-maternal outcomes. Cornual wedge resection and cornuostomy are the 2 main surgical management procedures for cornual pregnancy. According to the results of the retrospective studies, there was no significant difference in fertility and obstetrical outcomes between these 2 strategies.^[9,10]^

Presently, these are no distinctive and specific clinical symptoms to facilitate diagnosis of uterine rupture during gestation.^[8]^ Although the clinical manifestations of the injury vary, a sudden onset of abdominal pain, which happened to our patient, is one of the most common symptoms. Thus, differential diagnoses in the acute abdomen during pregnancy should include obstetrical and nonobstetrical etiologies. Ultrasound examination has potential advantages to evaluate the fetus and the pregnant uterus. It has high sensitivity and specificity to differentiate placenta implantation, concealed placental abruption, and urolithiasis. However, it has limitations in detecting acute appendicitis due to the unpredictable location of the appendix, which might be influenced by the enlarged pregnant uterus and intestinal pneumatosis. In addition, its utility in diagnosing uterine rupture is also limited. MRI has unique advantages in the differential workup of acute abdomen in hemodynamically stable pregnant patients and has demonstrated superior accuracy in the measurement of uterine wall defects.^[11,12]^ In our case, an MRI scan indicated that there was a 49 mm uterine wall dehiscence suspected to be a complete rupture of the myometrium scar with an intact amniotic sac protruding out of the peritoneal cavity.

Thus, rapid confirmation of a diagnosis is critical to initiate a prompt intervention for saving the lives of the fetus and the mother, once uterine rupture is suspected. An accurate diagnosis of uterine rupture relies on the history of uterine surgery, manifestations, physical examination, and imaging studies. In principle, termination of the pregnancy and repair of the defects should be considered immediately once gestational uterine rupture has been confirmed. Urgent cesarean delivery with either uterine repair or hysterectomy may be appropriate for fetal viability.

In conclusion, uterine rupture should be suspected when abdominal pain occurs in the last trimester with a history of laparoscopic hysterotomy. Swift diagnosis and prompt intervention contribute to improving fetal and maternal outcomes.

## Acknowledgment

The authors thank the patient for consenting to publish this case report.

## Author contributions

**Conceptualization:** Hong He.

**Data curation:** Guixian Chen, Yaoyue Zhang, Yuan Li, Yi Li.

**Investigation:** Guixian Chen, Yaoyue Zhang, Yuan Li, Yi Li.

**Material collecting and writing – editing:** Guixian Chen, Yaoyue Zhang, Yi Luan, and Yi Li.

**Supervision:** Hong He.

**Writing – original draft:** Jianyang Feng and Yahui Kang.

**Writing – review & editing:** Hong He.
